# One year in: early European adoption of the da Vinci Single Port platform—a multi‐institutional study from the European Association of Urology Robotic Urology Section Scientific Working Group

**DOI:** 10.1111/bju.70193

**Published:** 2026-02-25

**Authors:** Francesco Pellegrino, Antony Pellegrino, Ugo Giovanni Falagario, Marco Tozzi, Robin Weston, Vishwanath Hanchanale, Giorgio Gandaglia, Alberto Briganti, Francesco Montorsi, Gabriele Tuderti, Aldo Brassetti, Giuseppe Simone, Alessandro Izzo, Gianluca Spena, Roberto Contieri, Sisto Perdonà, Alessandro Tedde, Massimo Madonia, Stefano Tappero, Elio Mazzone, Paolo Dell'Oglio, Antonio Galfano, Karel Decaestecker, Peter Dekuyper, Filip Ameye, Federico Lavagno, Giancarlo Marra, Paolo Gontero, Carlo Costi, Giovanni Passaretti, Antonio Russo, Franco Gaboardi, Axel Möller, Fredrik Sundquist, Mats Olsson, Chiara Vaccaro, Francesco Alessandro Mistretta, Stefano Luzzago, Gennaro Musi, Andrea Piccolini, Marco Paciotti, Giovanni Lughezzani, Nicolò Maria Buffi, Enrico Checcucci, Paolo Alessio, Daniele Amparore, Francesco Porpiglia, Filippo Marino, Luca Di Gianfrancesco, Davide De Marchi, Angelo Porreca, Christian Fankhauser, Matteo Ferro, Alberto Breda, Simone Crivellaro, Peter Wiklund, ERUS Scientific Working Group

**Affiliations:** ^1^ Department of Urology ASST Santi Paolo e Carlo Milan Italy; ^2^ Unit of Urology/Division of Oncology, Urological Research Institute IRCCS San Raffaele Hospital Milan Italy; ^3^ Vita‐Salute San Raffaele University Milan Italy; ^4^ Department of Urology ASST Grande Ospedale Metropolitano Niguarda Milan Italy; ^5^ Department of Urology IRCCS San Raffaele Hospital Milan Italy; ^6^ Department of Urology European Institute of Oncology (IEO), IRCCS Milan Italy; ^7^ Department of Oncology and Hemato‐Oncology Università degli Studi di Milano Milan Italy; ^8^ Department of Biochemical Science Humanitas University Milan Italy; ^9^ Department of Urology Humanitas Clinical and Research Institute IRCCS Milan Italy; ^10^ Department of Urology University of Foggia Foggia Italy; ^11^ Department of Urology IRCCS “Regina Elena” National Cancer Institute Rome Italy; ^12^ Department of Urology, Istituto Nazionale Tumori di Napoli IRCCS, Fondazione “G. Pascale” Naples Italy; ^13^ Urology Department, Department of Medicine, Surgery and Pharmacy AOU Sassari Sassari Italy; ^14^ Division of Urology Molinette Hospital ‐ Città della Salute e della Scienza di Torino Turin Italy; ^15^ Department of Surgery, FPO‐IRCCS Candiolo Cancer Institute Candiolo, Turin Italy; ^16^ Department of Oncology, Division of Urology, San Luigi Gonzaga Hospital University of Turin Orbassano, Turin Italy; ^17^ Department of Urology, Humanitas Gavazzeni Humanitas Gavazzeni Bergamo Italy; ^18^ Department of Molecular Medicine and Surgery Karolinska Institutet Stockholm Sweden; ^19^ Royal Liverpool Hospital Liverpool University Hospitals NHS Foundation Trust Liverpool UK; ^20^ Department of Urology AZ Maria Middelares Ghent Belgium; ^21^ Centre for Integrative Genomics, Faculty of Biology and Medicine Genopode Building University of Lausanne Lausanne Switzerland; ^22^ Department of Urology, Fundació Puigvert Autonomous University of Barcelona Barcelona Spain; ^23^ Department of Urology University of Illinois at Chicago Chicago Illinois USA; ^24^ Icahn School of Medicine at Mount Sinai Health System New York City New York USA

**Keywords:** single‐port, robotic surgery, training, radical prostatectomy, partial nephrectomies

**Abbreviations**
SP: da Vinci Single Port
LND: lymph node dissection
RARP: robot‐assisted radical prostatectomy
RASP: robot‐assisted simple prostatectomy

The da Vinci Single Port™ (SP) robotic platform (Intuitive Surgical, Sunnyvale, CA, USA) received United States Food and Drug Administration (FDA) approval in 2018, marking a new chapter in robotic surgery through a single‐arm entry system with a reduced working envelope. Its approval in Europe in early 2024 introduced it into a competitive, rapidly evolving robotic landscape. Unlike the United States setting—where Intuitive holds a dominant monopoly—the European robotic market is characterised by multiplatform implementation, with systems such as the Medtronic HUGO™ Robot‐Assisted Surgery system (Medtronic Inc., Minneapolis, MN, USA) and CMR Versius® Robotic Surgical System (CMR Surgical, Cambridge, UK) already installed in several hospitals [1]. This broader adoption landscape brings new challenges and opportunities regarding technology dissemination, training, and clinical adaptation. Its limited working space and lack of triangulation compared to multiport platforms have led to the development of novel surgical techniques [2]. A key advantage is the possibility of an extraperitoneal/retroperitoneal route, expanding robotic indications to patients once considered inoperable due to prior surgery, hostile anatomy, or comorbidities [3].

In light of the recent European approval, the European Association of Urology Robotic Urology Section (ERUS) Working Group undertook a collaborative effort to assess real‐world patterns of SP adoption in Europe during its first year. A structured survey was distributed via Google Forms to all European centres known to have installed the SP platform between February 2024 and January 2025. The survey collected institutional and procedural data, including the number of SP and multiport cases per quarter. We reported the number of urological robot‐assisted procedures performed and the percentage carried out with the SP platform. Moreover, we plotted the proportion of the most common urological robot‐assisted procedures performed with the SP per quarter after its installation at each centre and tested whether there was a significant trend in SP utilisation using the Cochran–Armitage test.

Updated data were retrieved from 15 centres out of more than 20 that were eligible, offering a comprehensive snapshot of SP usage across Europe. Table S1 reports centre characteristics. All were high‐volume robotic hubs with at least one multiport platform before adopting the SP platform, which was used by one (one centre) to11 (one centre) urologists per centre. The multiport platform was often shared with other specialties (general surgery in 15 centres, cardiothoracic surgery in 11 centres, gynaecology in 10). The number of robotic urological procedures performed in 2024 ranged from 88 to 1400 cases per centre. Of these, SP procedures ranged from 11 to 200, representing between 4% and 67% of the robotic urologic procedures (Table 1). Usage varied by institution and indication, with some centres reporting SP utilisation rates up to 100% for specific operations (e.g., centre 15 for robot‐assisted simple prostatectomy [RASP]). In three centres, the SP was used exclusively by urologists. Most centres shared the SP platform with other departments such as general surgery (11 centres) and gynaecology (seven centres), indicating early cross‐specialty integration.

**Table 1:** The SP activities across centres.

Centres	Urologists using SP, *n*	SP procedures*, *n* (%)	RARP no LND*, *n* (%)	RARP + LND*, *n* (%)	RASP*, *n* (%)	RAPN*, *n* (%)	RA radical nephrectomy*, *n* (%)	RA NUT*, *n* (%)	RA pyeloplasties*, *n* (%)	RARC*, *n* (%)	Other SP procedures, *n* (%)	Other specialists using SP, *n* (%)
Centre 1	2	28 (7)	28 (21)	0 (0)	–	0 (0)	0 (0)	0 (0)	0 (0)	0 (0)		General surgery
Centre 2	4	20 (4)	15 (7)	0 (0)	1 (10)	1 (1)	0 (0)	0 (0)	1 (1)	0 (0)		No
Centre 3	4	150 (11)	55 (39)	0 (0)	5 (20)	60 (21)	0 (0)	0 (0)	0 (0)	0 (0)		Cardiothoracic surgery, Gynaecology, Head and Neck Surgery
Centre 4	3	65 (14)	19 (31)	6 (5)	–	22 (14)	3 (16)	8 (50)	–	5 (6)	RPLND 2 (40)	Cardiothoracic surgery, General surgery, Gynaecology, Head and Neck Surgery
Centre 5	1	28 (32)	12 (70)	5 (17)	–	2 (10)	–	–	5 (83)	4 (33)		General Surgery, Gynaecology
Centre 6	7	47 (11)	27 (27)	2 (2)	3 (43)	17 (18)	2 (7)	5 (23)	10 (27)	1 (4)		General Surgery, Gynaecology, Head and Neck Surgery
Centre 7	3	121 (36)	32 (21)	0 (0)	1 (50)	35 (62)	5 (24)	4 (21)	11 (69)	0 (0)	Colpo‐promontorium fixation 4 (20), Kidney transplantation 3 (27)	Cardiothoracic surgery, General surgery
Centre 8	1	56 (17)	17 (13)	2 (1)	1 (100)	1 (2)	2 (100)	14 (82)	8 (67)	0 (0)		General Surgery
Centre 9	3	11 (7)	0 (0)	7 (6)	–	2 (8)	1 (100)	–	1 (67)	0 (0)		No
Centre 10	8	91 (19)	81 (36)	4 (9)	0 (0)	8 (100)	1 (100)	–	2 (100)	3 (2)		General surgery, Gynaecology, Head and Neck Surgery
Centre 11	7	66 (8)	45 (57)	6 (1)	1 (5)	10 (5)	0 (0)	2 (9)	–	0 (0)	Inguinal LND 2 (100)	Cardiothoracic surgery, General surgery, Gynaecology, Head and Neck Surgery
Centre 12	2	14 (3)	6 (4)	0 (0)	4 (100)	3 (3)	0 (0)	0 (0)	1 (5)	0 (0)		General surgery
Centre 13	2	50 (33)	25 (62)	5 (7)	10 (100)	10 (4)	2 (11)	2 (20)	1 (20)	0 (0)		General surgery, Gynaecology
Centre 14	2	36 (22)	27 (61)	2 (4)	1 (100)	3 (9)	0 (0)	0 (0)	1 (33)	1 (9)		No
Centre 15	4	200 (67)	40 (80)	90 (90)	10 (100)	30 (100)	2 (7)	15 (100)	15 (100)	23 (92)	RPLND 15 (100)	General surgery
**Overall**		983 (15)	429 (27)	129 (7)	37 (41)	204 (17)	18 (4)	50 (18)	56 (23)	37 (5)		

Percentages in parentheses indicate the proportion of SP procedures relative to the total number of that procedure performed at the respective centre. NUT, nephroureterectomy; RA, robot‐assisted; RAPN, robot‐assisted partial nephrectomy; RARC, robot‐assisted radical cystectomy; RPLND, retroperitoneal lymph node dissection.

* Number of procedures performed with the SP platform between February 2024 and January 2025 in each centre.

The most frequent urological procedure performed was robot‐assisted radical prostatectomy (RARP) with lymph node dissection (LND) (1761 cases); 129 were performed with the SP (7%) (Table 1). The most common procedure performed with the SP was RARP without LND (429 cases), accounting for 27% of all RARPs without LND. Similarly, 17% of robot‐assisted partial nephrectomies and 23% of robot‐assisted pyeloplasties were performed with the SP. By contrast, only 5% of the robot‐assisted radical cystectomy were performed with the SP.

Figure S1 shows SP utilisation trends by quarter (two centres were excluded for missing data). Utilisation rose for all procedures (RARP from 20% to 37%, nephrectomy from 16% to 25%, RASP from 24% to 59%, and reconstructive surgery from 56% to 72%), reaching statistical significance only for RARP and nephrectomy (*P* < 0.01).

These data confirm that, despite its recent market entry, the SP platform is already making a tangible impact on surgical practice in Europe. Differences from United States adoption patterns are evident and may be explained by the greater procedural flexibility afforded by European surgical training pathways, fewer formal credentialing barriers, and a more competitive robotic platform environment. Importantly, the versatility of the SP has led to a remodelling of surgical indications and approaches [2]. Extraperitoneal and retroperitoneal techniques are increasingly favoured, offering solutions for complex patients previously relegated to open surgery. In addition, the platform also enables outpatient pathways, already established in the United States and potentially transferable to Europe, although further studies are needed to confirm feasibility and safety [4, 5].

Of particular interest is the relatively high rate of SP adoption in RASP. This may stem from the historically low uptake of RASP with multiport systems in Europe and the technical advantages of the SP for the transvesical route. This approach is technically easier to execute using the SP due to its linear access and reduced arm collision. Furthermore, in many European centres, tutorship during SP case initiation was provided by visiting United States‐based urologists who had early experience with the SP and preferentially employed it for RASP, given its rapid learning curve and low complication rate [6]. This transatlantic mentorship likely contributed to the early inclusion of RASP in SP programmes, despite its relatively low baseline frequency in European practice.

Another notable finding is the high proportion of SP RARP performed without LND and the very low rate of cases including LND. This reflects a tendency to preferentially perform RARP with LND using multiport systems, as these platforms are perceived to better ensure oncological radicality and adequate nodal yield. Similarly, the limited number of cystectomies performed with the SP can be explained by the technical challenges of applying an extraperitoneal approach to such complex operations, although some pioneering centres are actively exploring this indication. In both settings, the main advantage of the SP—its capacity to facilitate extraperitoneal surgery—may paradoxically restrict its use.

Moreover, the recent Conformité Européenne (CE)‐mark approval of the SHURUI® single‐port surgical robot (Beijing Surgerii Robotics Company Ltd., Beijing, China) may further shape the European landscape, potentially influencing future adoption trends, competition, and platform diversification in the single‐port space.

This study is not without limitations. Not all eligible centres participated in the survey; however, data were captured from the majority of institutions (15 of ~20). In addition, not all centres had possessed the SP platform for at least 1 year, meaning that adoption trends by trimester—particularly in the third and fourth—do not include the full complement of centres.

Overall, these findings underscore the transformational potential of the SP in both broadening the indications for robotic surgery and reshaping traditional operative algorithms. Future studies should assess outcomes, learning curves, ergonomics, and cost‐effectiveness to define which patients and procedures benefit most from the SP and how best to train surgical teams across platforms.

## Funding

This research received no specific grant from any funding agency in the public, commercial, or not‐for‐profit sectors.

## Disclosure of Interests

The authors declare no conflicts of interest in preparing this article.

## Supporting information


**Table S1.** Centre characteristics.
**Fig. S1**. Number of the most common robot‐assisted urological procedures performed with the da Vinci SP (red) or multiport platforms (blue) per quarter following installation of the da Vinci SP at each centre. Percentages indicate the proportion of SP procedures relative to the total number of that procedure performed in that quarter.
