# Supplementary material for: One year in: early European adoption of the da Vinci Single Port platform—a multi‐institutional study from the European Association of Urology Robotic Urology Section Scientific Working Group
Source: BJU Int. 2026 Feb 25;137(6):975–8. doi: 10.1111/bju.70193 (PMC13168928; doi:10.1111/bju.70193)
Supplement: Supplementary file 1 — Table S1. Centre characteristics. Fig. S1. Number of the most common robot‐assisted urological procedures performed with the da Vinci SP (red) or multiport platforms (blue) per quarter following installation of the da Vinci SP at each centre. Percentages indicate the proportion of SP procedures relative to the total number of that procedure performed in that quarter. [file BJU-137-975-s001.docx]

**Supplementary material**

**- sTable 1**. Center characteristics

**- sFigure 1.** Number of the most common robot-assisted urologic procedures performed with the Da Vinci Single Port (red) or multiport systems (blue) per quarter following installation of the Da Vinci Single Port at each center. Percentages indicate the proportion of Single Port procedures relative to the total number of that procedure performed in that quarter

**sTable 1**. Center characteristics

| **Centers** | **Country** | **SP Introduction Date** | **N° RA Procedures Feb-24 Jan-25** | **Robotic Platforms Before SP** | **N° Urologists Using Robotic Platforms** | **Other Specialists Using Robotic Platforms** |
| --- | --- | --- | --- | --- | --- | --- |
| Center 1 | UK | 19/11/24 | 425 | Da Vinci Xi and X | 5 | Cardiothoracic surgery, General Surgery, Gynecology |
| Center 2 | Italy | 01/06/24 | 500 | Da Vinci Xi and X | 10 | Cardiothoracic surgery, General surgery |
| Center 3 | Italy | 18/05/24 | 1400 | Da Vinci Xi | 10 | Cardiothoracic surgery, General Surgery, Gynecology, Head and Neck Surgery |
| Center 4 | Italy | 07/05/24 | 467 | Da Vinci Xi and X | 10 | Cardiothoracic surgery, General surgery, Gynecology, Head and Neck Surgery |
| Center 5 | Italy | 16/09/24 | 88 | Da Vinci Xi | 1 | General Surgery, Gynecology |
| Center 6 | Italy | 08/05/24 | 426 | Da Vinci Xi  Hugo RAS | 10 | General Surgery, Gynecology, Head and Neck Surgery |
| Center 7 | Belgium | 22/05/24 | 505 | Da Vinci Xi and X  Hugo RAS | 5 | Cardiothoracic surgery, General Surgery, Gynecology |
| Center 8 | Italy | 26/06/24 | 333 | Da Vinci Xi | 7 | Cardiothoracic surgery, General Surgery |
| Center 9 | Italy | 01/06/24 | 150 | Da Vinci Xi and X  Hugo RAS | 5 | Cardiothoracic surgery, General Surgery, Head and Neck Surgery |
| Center 10 | Sweden | 04/04/24 | 476 | Da Vinci Xi and X  Hugo RAS | 10 | General Surgery, Gynecology, Head and Neck Surgery, Pediatric surgery |
| Center 11 | Italy | 27/06/24 | 824 | DaVinci Xi | 11 | Cardiothoracic surgery, General surgery, Gynecology, Head and Neck Surgery, Senology |
| Center 12 | Italy | 07/10/24 | 439 | Da Vinci X and Xi | 5 | Cardiothoracic surgery, General surgery, Gynecology, Head and Neck Surgery |
| Center 13 | Italy | 01/07/24 | 150 | Da Vinci Xi | 3 | General surgery, Gynecology |
| Center 14 | Italy | 25/09/24 | 167 | DaVinci Xi | 3 | Cardiothoracic surgery, General Surgery |
| Center 15 | Switzerland | 07/04/24 | 300 | DaVinci Xi | 4 | Cardiothoracic surgery, General surgery, Head and Neck Surgery |

SP: Da Vinci Single Port, RA: robot-assisted

**sFigure 1.** Number of the most common robot-assisted urologic procedures performed with the Da Vinci Single Port (red) or multiport systems (blue) per quarter following installation of the Da Vinci Single Port at each center. Percentages indicate the proportion of Single Port procedures relative to the total number of that procedure performed in that quarter
